# 154. Pharmacokinetics and Safety of GS-1720 Following Multiple Ascending Doses in a Phase 1a Study in People Without HIV-1

**DOI:** 10.1093/ofid/ofae631.040

**Published:** 2025-01-29

**Authors:** Haeyoung Zhang, Ines Mendes, Eva Mortensen, Hui Wang, Aaron Share, Monika Sobczyk, Ramesh Palaparthy, Dhananjay Marathe

**Affiliations:** Gilead Sciences Inc, Foster City, California; Gilead Sciences, Inc., Foster City, California; Gilead Sciences Inc, Foster City, California; Gilead Sciences Inc., Foster City, California; Gilead Sciences, Inc., Foster City, California; Gilead Sciences, Inc., Foster City, California; Gilead Sciences Inc, Foster City, California; Gilead Sciences, Inc., Foster City, California

## Abstract

**Background:**

Long-acting antiretrovirals for HIV-1 treatment may improve adherence by reducing treatment fatigue. GS-1720 is an oral integrase strand transfer inhibitor (INSTI) with potent anti-HIV-1 activity. Pharmacokinetics (PK) and safety of single ascending GS-1720 doses in participants without HIV-1 have been previously reported. Here, we report preliminary PK and safety of multiple ascending doses (MAD).

**Methods:**

In this Phase 1a, randomized, blinded, placebo-controlled, multicohort study, oral GS-1720 or placebo (6:3/cohort) was administered under fasting conditions in 3 once-weekly (QW) and 2 once-daily (QD) MAD cohorts. Participants received 150, 450, or 1350 mg of GS-1720, or placebo, for 6 weeks or 450 or 900 mg of GS-1720, or placebo, for 7 or 14 days, respectively. Primary endpoints were plasma PK parameters (including AUC, C_max_, and accumulation ratios) and incidence of adverse events (AEs) and laboratory abnormalities. Participants were followed up to Day (D) 105.

**Results:**

Overall, 45 participants were enrolled: median age, 29–40 years; 47% female. For QW doses of 150, 450, or 1350 mg at Week 6, respectively, mean (coefficient of variation [CV]%) C_max_ was 19.9 (21.3), 39.6 (23.3), and 60.6 (25.8) μg/mL, and median (range) T_max_ was 6 (4–8), 4 (3–4), and 4 (2–6) hours. Accumulation ratios ranged from 2.4–2.9 for AUC and 2.0–2.2 for C_max_. For QD doses of 450 and 900 mg at D7 and D14, respectively, mean (CV%) C_max_ was 64.5 (13.6) and 126 (13.6) μg/mL, and median (range) T_max_ was 4 (2–8) hours. Accumulation ratios were 5.3 and 6.8 for AUC and 4.2 and 5.4 for C_max_ at D7 and D14, respectively. AEs related to GS-1720/placebo (blinded data) were reported in 12/45 participants (27%); most AEs (16/20; 80%) were Grade 1. One AE in the 450 mg QW cohort led to GS-1720/placebo discontinuation.

**Conclusion:**

GS-1720 exhibits a PK and safety profile supportive of oral QW dosing. The data support the development of GS-1720 as part of a first INSTI-containing oral QW regimen.

**Disclosures:**

**Haeyoung Zhang, PharmD, PhD**, Gilead Sciences, Inc.: Employee|Gilead Sciences, Inc.: Stocks/Bonds (Public Company) **Ines Mendes, PharmD**, Gilead Sciences, Inc.: Employee|Gilead Sciences, Inc.: Stocks/Bonds (Public Company) **Eva Mortensen, MD, MS**, Gilead Sciences, Inc.: Employee|Gilead Sciences, Inc.: Stocks/Bonds (Public Company) **Hui Wang, PhD**, Gilead Sciences, Inc.: Employee|Gilead Sciences, Inc.: Stocks/Bonds (Public Company) **Aaron Share, B.Sc**, Gilead Sciences, Inc.: Employee|Gilead Sciences, Inc.: Stocks/Bonds (Public Company) **Monika Sobczyk, M.Sc**, Gilead Sciences, Inc.: Employee|Gilead Sciences, Inc.: Stocks/Bonds (Public Company) **Ramesh Palaparthy, PhD**, Gilead Sciences, Inc.: 1475-US-PSP, 1475-WO-PCT, 1474-US-PSP, 1515-US-PSP, 1515-WO-PCT|Gilead Sciences, Inc.: Employee|Gilead Sciences, Inc.: Stocks/Bonds (Public Company) **Dhananjay Marathe, PhD**, Gilead Sciences, Inc.: Employee|Gilead Sciences, Inc.: Stocks/Bonds (Public Company)

